# Supplementary material for: Cell competition promotes metastatic intestinal cancer through a multistage process
Source: iScience. 2024 Apr 11;27(5):109718. doi: 10.1016/j.isci.2024.109718 (PMC11068562; doi:10.1016/j.isci.2024.109718)
Supplement: Document S1. Figures S1–S6 and Table S1 [file mmc1.pdf]

iScience, Volume *27*

Supplemental information

Cell competition promotes metastatic intestinal cancer through a multistage process

Ana Krotenberg Garcia, Mario Ledesma-Terrón, Maria Lamprou, Joyce Vriend, Merel Elise van Luyk, and Saskia Jacoba Elisabeth Suijkerbuijk

Figure S1

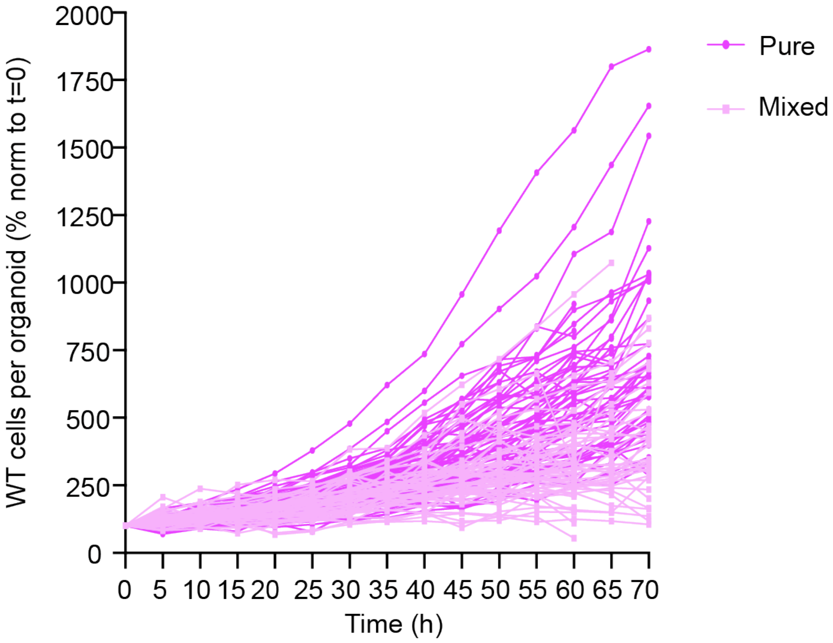

Figure S1: Cancer cells outcompete wild-type liver cells

Related to Figure 1

Displays the wild-type cells in pure (dark purple) and mixed (light purple) that were followed during time-lapse imaging. The number of wild-type cells, normalized to T=0, is plotted against time. Each line represents an organoid.

Figure S2

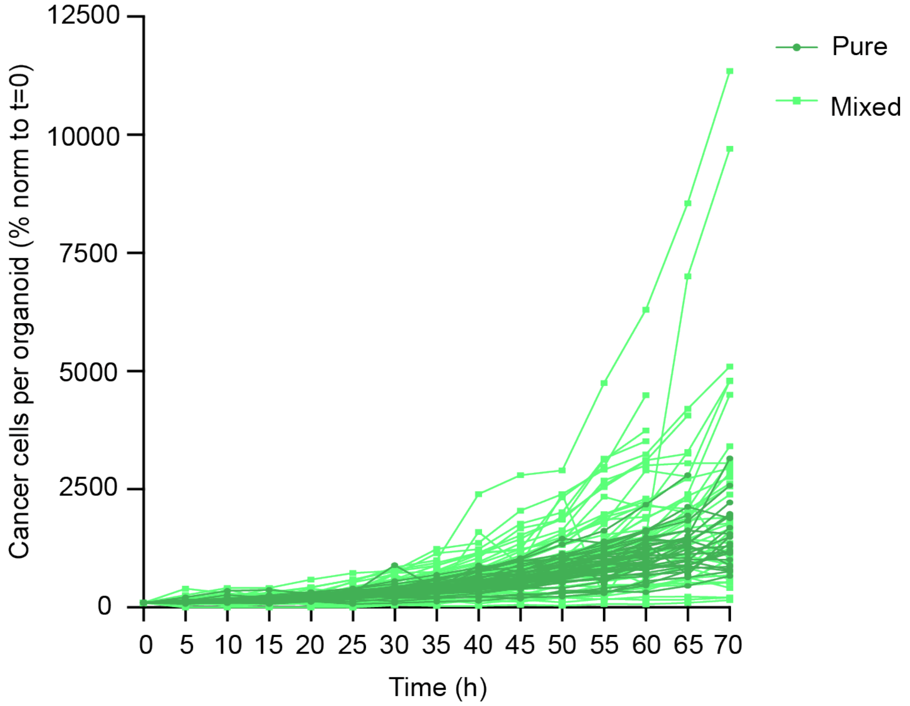

Figure S2: Increased expansion of competing cancer cells

Related to Figure 2

Displays the cancer cells in pure (dark green) and mixed (light green) that were followed during time-lapse imaging. The number of cancer cells, normalized to T=0, is plotted against time. Each line represents an organoid.

Figure S3

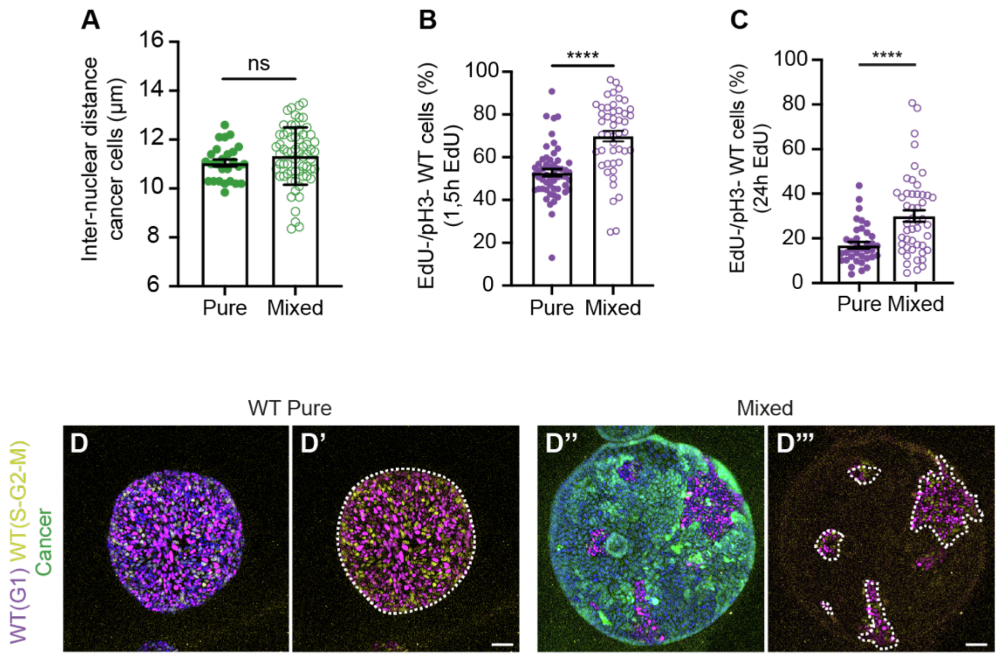

Figure S3: Cancer induces compaction and cell cycle arrest of wild-type liver cells

Related to Figure 3

(A) Quantification of the inter-nuclear distance of cancer cells in pure and mixed organoids; each dot represents one organoid (mean ± SEM; Ordinary one-way ANOVA, Sidak's multiple comparisons test; p<0.4681; n= 26 and 64 organoids). (B-C) Quantification of the percentage of EdU-/pH3- wild-type cells after 1.5 h (B) or 24 h (C) of EdU treatment; each dot represents one organoid (mean ± SEM; Ordinary one-way ANOVA, Sidak's multiple comparisons test; p<0.0001; n= 55 and 47 organoids in B; p<0.0001; n=37 and 48 organoids in C). (D) Z projection confocal images of pure wild-type (D and D') and Mixed (D'' and D''') FUCCI2 organoids fixed three days after planting. White dotted line indicates wild-type cells.

Scale bars represent 50 µm.

Figure S4

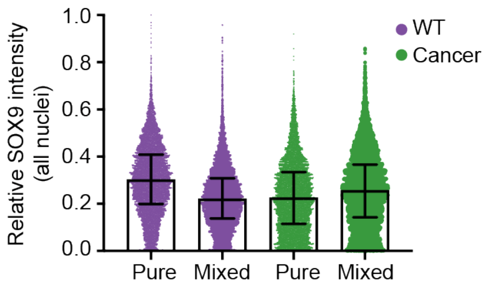

Figure S4: Increased competition through loss of the WT progenitor state

Related to Figure 4

Displays the relative SOX9 intensities of all individual nuclei in pure and mixed organoids; each dot represents a nucleus (WT pure median =0.2475, n= 7998 nuclei; WT mixed median = 0.1870, n= 9083 nuclei; cancer pure median= 0.2686, n= 8093 nuclei; cancer mixed median= 0.2880, n= 66127).

Figure S5

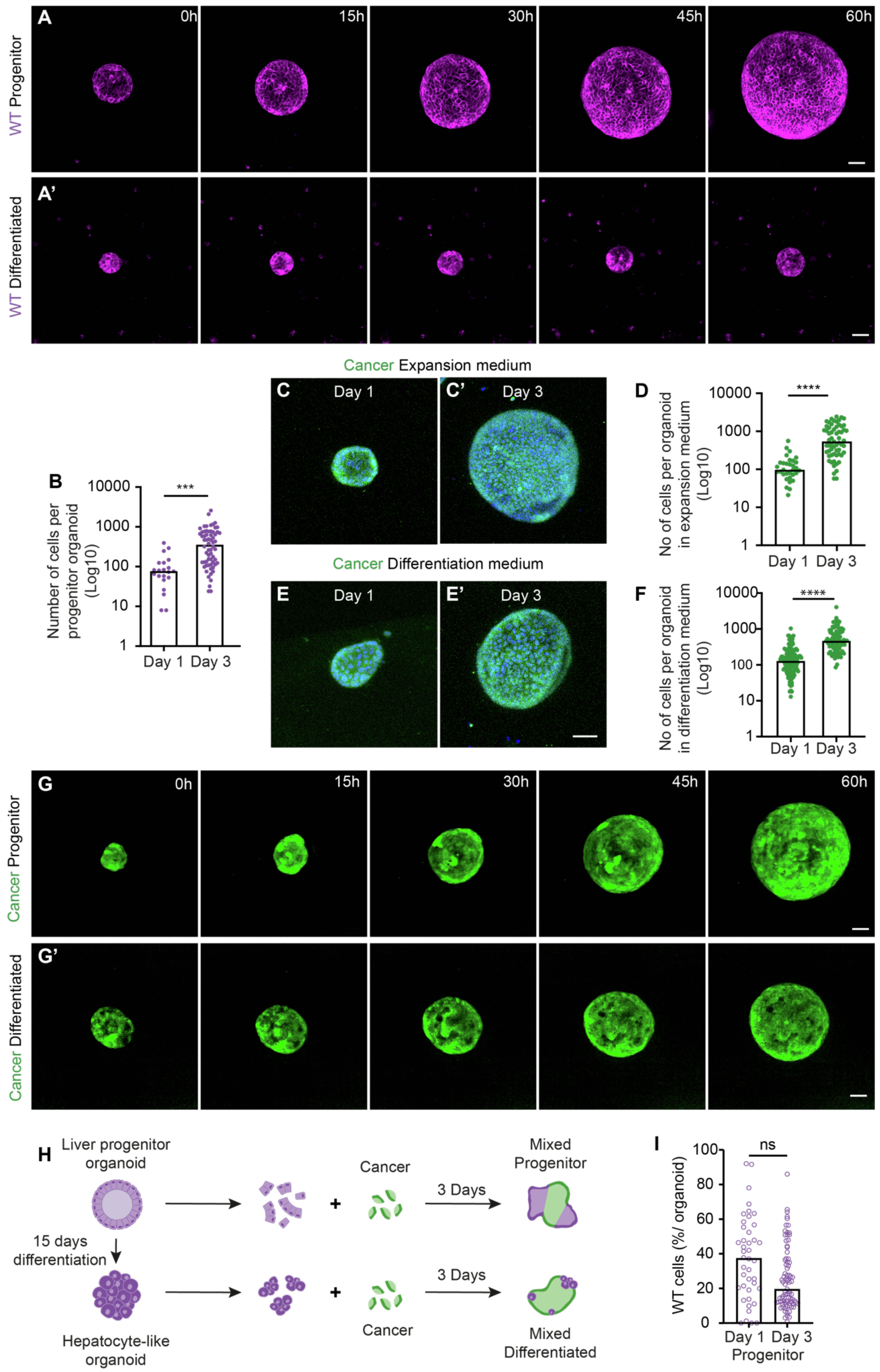

A
0h
15h
30h
45h
60h
WT Progenitor
A'
WT Differentiated
Cancer Expansion medium
C
Day 1
C'
Day 3
D
No of cells per organoid in expansion medium (Log10)
10000
1000
100
10
1
****
Day 1
Day 3
B
Number of cells per progenitor organoid (Log10)
10000
1000
100
10
1
***
Day 1
Day 3
Cancer Differentiation medium
E
Day 1
E'
Day 3
F
No of cells per organoid in differentiation medium (Log10)
10000
1000
100
10
1
****
Day 1
Day 3
G
0h
15h
30h
45h
60h
Cancer Progenitor
G'
Cancer Differentiated
H
Liver progenitor organoid
15 days differentiation
Hepatocyte-like organoid
Cancer
3 Days
Mixed Progenitor
3 Days
Cancer
Mixed Differentiated
I
WT cells (%/ organoid)
100
80
60
40
20
0
ns
Day 1
Day 3
Progenitor

Figure S5 - Differentiated WT cells are effectively outcompeted

Related to Figure 5

(A) Representative maximum projections of 3D-confocal images of time-lapse series of WT progenitor (A) and WT differentiated (A') cells. (B) Quantification of the number of WT progenitor cells; each dot represents one organoid (Median; Kruskal-Wallis test, Dunn´s multiple comparison test; $p=0.0008$; $n=21$ and $n=64$). (C-F) Representative maximum projections of 3D-confocal images of pure cancer organoids cultured in expansion (C) and differentiation (E) medium, fixed at day 1 (C and E) and day 3 (C' and E') after plating; nuclei are visualized with DAPI (blue). Graphs display the number of cancer cells in pure cancer organoids cultured in expansion (D) and differentiation (F) medium; each dot represents one organoid (Median; Kruskal-Wallis test, Dunn´s multiple comparison test; $p<0.0001$; $n=32$ and $n=61$, D; $n=124$ and $n=87$, F). (G) Representative maximum projections of 3D-confocal images of time-lapse series of pure cancer organoids cultured in progenitor (G) and differentiation (G') medium. (H) Schematic depiction of the generation of mixed organoids from progenitor cholangiocyte (top) and differentiated hepatocyte-like (bottom) organoids. (I) shows the percentage of WT progenitor cells in mixed organoids at day 1 and day 3; each dot represents one organoid (Median; Kruskal-Wallis test, Dunn´s multiple comparison test; $p=0.1671$; $n=42$ and $n=81$).

Scale bars represent 50 µm.

Figure S6

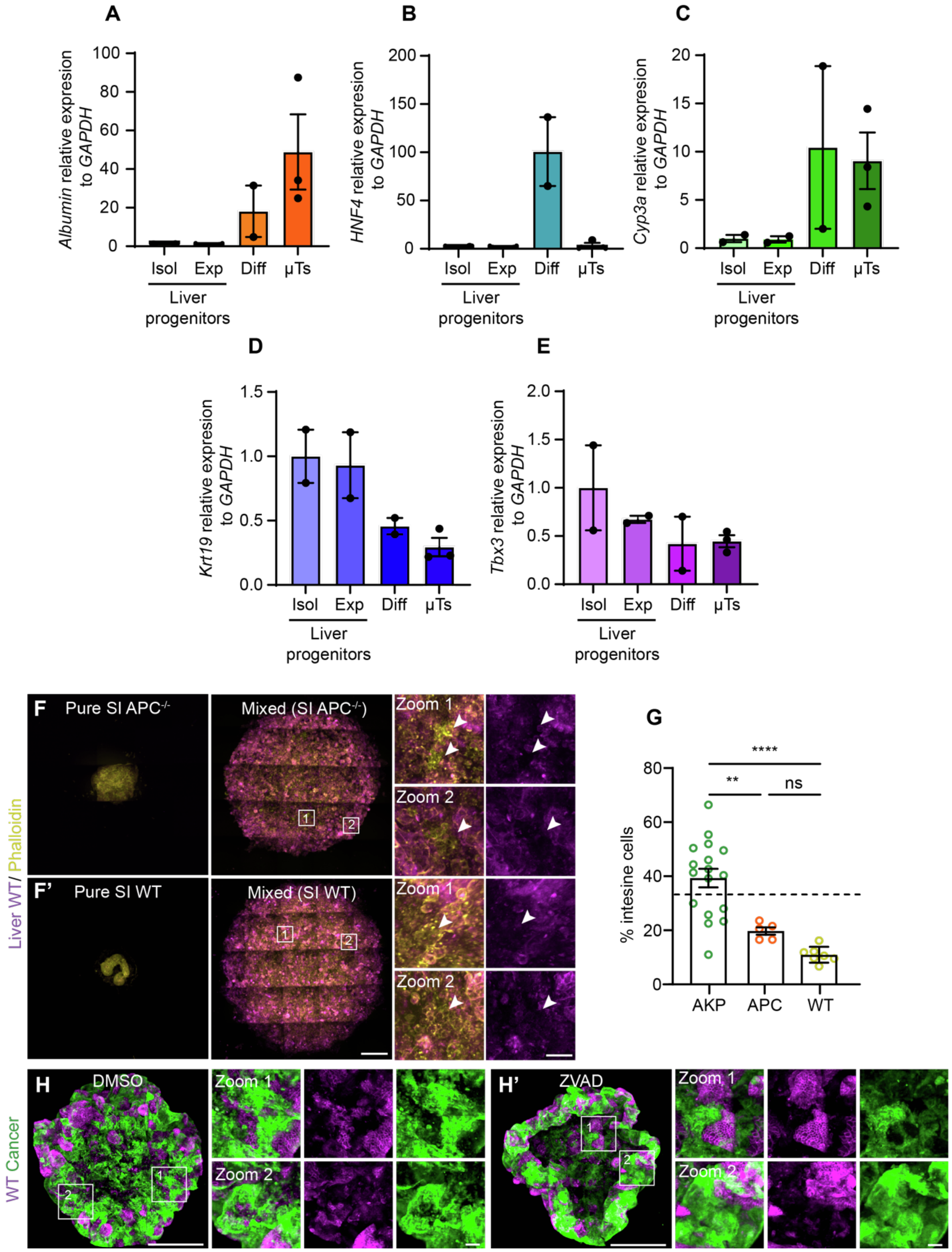

A
B
C
Albumin relative expresion to GAPDH
HNF4 relative expresion to GAPDH
Cyp3a relative expresion to GAPDH
Isol
Exp
Diff
µTs
Liver progenitors
D
E
Krt19 relative expresion to GAPDH
Tbx3 relative expresion to GAPDH
F
Pure SI APC-/-
Mixed (SI APC-/-)
Zoom 1
Zoom 2
F'
Pure SI WT
Mixed (SI WT)
Liver WT/ Phalloidin
G
% intesine cells
****
**
ns
AKP
APC
WT
H
DMSO
H'
ZVAD
WT Cancer

Figure S6 - WT liver acts as a scaffold for tumor growth during competition
Related to Figure 6

(A-E) Display the expression of hepatocyte marker genes *Albumin* (A), *HNF4* (B) and *Cyp3a* (C) and liver progenitor marker genes *Krt19* (D) and *Tbx3* (E) relative to the housekeeping gene *GAPDH*, in liver progenitor organoids cultured for 3 days in isolation and expansion medium, in liver progenitor organoids at the day 15 of the differentiation protocol and in microtissues 3 days after plating. (F) Representative 3D-reconstructed stitched confocal images of pure (left) and mixed (right) microtissues formed from $APC^{-/-}$ (F) and wild-type small intestine (F') organoids together with hepatocyte-like WT cells (Magenta). The insets display a 6x magnification of the area in the white box. (G) Quantification of the percentage of intestine cells in the mixed AKP, $APC^{-/-}$ and wild-type microtissues. The dashed line indicates the expected contribution based on the starting ratio (Mean; Ordinary one-way ANOVA, multiple comparisons; Tukey´s multiple comparison test; AKP vs APC, $p= 0.0049$, $n= 17$ and $n= 5$; AKP vs SI WT, $p<0.0001$, $n= 17$ and $n= 7$; APC vs SI WT $p= 0.4293$, $n= 5$ and $n= 7$). (H) Representative 3D-reconstructed stitched confocal images of mixed microtissues formed from AKP cancer (green) and hepatocyte-like WT cells (magenta), treated with vehicle control (DMSO) or pan-caspase inhibitor (ZVAD). The insets display a 2.5x magnification of the area in the white boxes. Scale bar in stitched overview image represent 500 µm and in the magnification 50 µm.

Table S1

Primer ID	Sequence
Albumin (mouse) FWD	AGCCCACTGTCTTAGTGAGG
Albumin (mouse) RV	TCTTGCACACTTCCTGGTCC
HNF4 (mouse) FWD	GCTAAGGCGTGGGTAGGG
HNF4 (mouse) RV	AGGCTGTTGGATGAATTGAGG
Cyp3a (mouse) FWD	TGGTCAAACGCCTCTCCTTGCT
Cyp3a (mouse) RV	ACTGGGCCAAAATCCCGCCG
Keratin 19 (mouse) FWD	GTCCTACAGATTGACAATGC
Keratin 19 (mouse) RV	CACGCTCTGGATCTGTGACA
Tbx3 (mouse) FWD	GAGGCCAAGGAACTTTGGGA
Tbx3 (mouse) RV	AGGGAACATTCGCCTTCCTG
GAPDH (mouse) FWD	ATGGTGAAGGTCGGTGTGAAC
GAPDH (mouse) RV	GCCGTGAGTGGAGTCATACTG

Table S1

Related to Figure S6

Overview of all oligonucleotide sequences used for qPCR experiments in Figure S6.
